# Global prevalence and risk factors of obstetric violence: A systematic review and meta‐analysis

**DOI:** 10.1002/ijgo.16145

**Published:** 2025-01-13

**Authors:** Sevil Hakimi, Leila Allahqoli, Maryam Alizadeh, Meryem Ozdemir, Hamid Soori, Esin Ceber Turfan, Neriman Sogukpinar, Ibrahim Alkatout

**Affiliations:** ^1^ Faculty of Health Sciences EGE University Izmir Turkey; ^2^ Ministry of Health and Medical Education Tehran Iran; ^3^ Tabriz University of Medical Science, Myianeh Branch Myianeh Iran; ^4^ Çiğli Educational and Training Hospital University of Bakirçay Izmir Turkey; ^5^ Faculty of Medicine Cyprus International University Nicosia Northern Cyprus; ^6^ Department of Obstetrics and Gynecology Kiel School of Gynaecology Endoscopy Kiel Germany

**Keywords:** childbirth, non‐consented care, obstetric violence, respectful maternal care, vaginal delivery

## Abstract

**Background:**

Obstetric violence (OBV), defined as mistreatment or abuse during childbirth, is a pervasive global issue, albeit with regional differences, affecting women's physical and emotional well‐being.

**Objectives:**

The purpose of this systematic review and meta‐analysis is to assess the prevalence of OBV to identify risk factors associated with OBV and to make suggestions for improving maternal healthcare practices and policies.

**Search Strategy:**

In a systematic review and meta‐analysis, we searched four electronic databases for studies published over 10 years up to 31 January 2024: Medline (PubMed), Scopus, Embase, and Web of Science (WOS). The search was conducted among English language papers using a carefully curated set of keywords.

**Selection Criteria:**

We conducted a comprehensive review, including all observational reporting data on the prevalence of and risk factors associated with OBV, irrespective of geographical location. The studies included in the review were required to be published in peer‐reviewed. journals and available in the English language.

**Data Collection and Analysis:**

The data of the studies were summarized in an Excel file (version 19) and analyzed using R (version 4.2.3). A meta‐analysis was performed to evaluate the pooled prevalence of and identify risk factors associated with OBV.

**Main Results:**

The global prevalence of OBV estimated based on 25 studies, calculated with a random‐effects model, was 59% (95% confidence interval [CI] 0.48–0.70; *I* ^2^ = 99.5%). The most prevalent subdomain of OBV was non‐consented care (37%; 95% CI 0.23–0.50; *I* ^2^ = 99.7%). The following factors were found to be significantly associated with OBV: the presence of a midwife as skilled personnel beside the woman during childbirth (odds ratio [OR] [95% CI] = 0.4 [0.2–0.9]), which might reduce the likelihood of OBV; middle and high levels of income (OR [95% CI] = 0.5 [0.2–0.7]), which might also reduce the likelihood of OBV; and vaginal delivery (OR [95% CI] = 2.08 [1.1–3.08]), which is liable to increase the likelihood of OBV.

**Conclusion:**

This systematic review and meta‐analysis highlights the considerable prevalence and multifaceted nature of OBV, underscoring the urgent need for interventions at multiple levels to address this pervasive issue and ensure respectful, safe, and dignified maternal healthcare for all women.

## Synopsis

The study found a global obstetric violence prevalence of 55%, with non‐consented care being the most common form (33%). Factors reducing obstetric violence include midwife presence and higher income levels, while vaginal delivery increases the likelihood of obstetric violence.

## 1 INTRODUCTION

Despite significant advancements in maternal care, reproductive health inequality remains unresolved in many countries.^1^ The World Health Organization (WHO) reports that many women experience disrespect and mistreatment during childbirth in healthcare facilities.^2^ Obstetric violence (OBV) refers to mistreatment and abuse endured by women during childbirth, which violates their rights, dignity, and autonomy.^3^ Such mistreatment might include forced medical procedures, non‐consented interventions, disrespectful language, neglect, and discrimination based on socioeconomic status, ethnicity or gender identity. It occurs in hospitals, clinics, and birthing centers. OBV types are context specific, with forced cesarean sections and frequent episiotomies prevalent in the Americas and Europe, while unattended childbirth in healthcare centers is more common in lower‐ and middle‐income countries.^4^ Some healthcare professionals reject the term OBV, denying that violence occurs during childbirth.^5^ The WHO acknowledges this issue as a violation of women's rights and prefers terms like “disrespect and abuse” or “mistreatment”.^6^ Critics argue that using the term OBV might put healthcare providers to shame and violate their rights.^7^ Public health authorities now emphasize not just childbirth outcomes but also the quality of maternal healthcare experiences.^8^, ^9^ The WHO recommends incorporating women's experiences into maternal health indicators.^10^, ^11^ Addressing OBV is crucial because it relates to human rights violations and negative maternal health outcomes such as post‐traumatic stress disorder and postpartum depression, eroding trust in healthcare systems.^12^, ^13^ Factors influencing OBV include marital status, maternal age, education level, socioeconomic status, race, parity, history of stillbirth or miscarriage, type of delivery, childbirth preparedness, anesthesia used, and whether the facility is public or private.^13^ Pre‐delivery preparedness programs and mother–infant contact after childbirth might afford protection against midwife violence.^3^ OBV can affect emotional and sexual relationships, bonding between mother and child, and breastfeeding.^14^, ^15^ It is not limited to developing countries; developed and high‐income countries are also affected.^16^ In Latin America, 25% of women experienced mistreatment during childbirth.^4^ The incidence of OBV varies globally (12%–75%), indicating cultural and national differences.^3^ Twenty‐four percent of women in Mexico reported maternal violence,^17^ while 17% of pregnant women in the USA were reported to experience OBV.^18^ Prevalence studies in Ghana, Guinea, Nigeria, and Myanmar yielded physical OBV rates of 37%, 36%, 49%, and 21%, respectively.^19^ OBV is manifested in different ways in Europe: high rates of episiotomies and dissatisfaction were reported in France and significant abuse in Italy.^20^

Despite the increasing attention to OBV in recent literature, there remains a lack of a comprehensive and unified figure that encapsulates the full scope of OBV based on existing evidence. Many studies focus on specific aspects or populations, leading to fragmented insights that do not provide a holistic view of the issue. This gap in the literature limits our understanding of the true prevalence and nature of OBV across different contexts. By synthesizing available data, this study aims to create a clearer picture of OBV, highlighting the need for robust methodologies and standardized definitions in future research. Establishing a complete figure of OBV is essential not only for raising awareness but also for guiding effective interventions and policy changes aimed at improving maternal healthcare experiences worldwide.

## 2 MATERIALS AND METHODS

The study protocol was prospectively registered at the International Prospective Register of Systematic Reviews (PROSPERO) and assigned the PROSPERO ID CRD42024504249. The systematic review and meta‐analysis adhered to the guidelines outlined in the Preferred Reporting Items for Systematic Reviews and Meta‐Analyses (PRISMA) framework.^21^ The research questions were formulated using the PICO framework. The key aspect addressed was the prevalence of OBV among women who had given birth. As no intervention or comparator was used, the PICO framework was focused solely on the target population (women who had given birth) and the outcome of interest (prevalence and related factors of OBV).

### 2.1 Search strategy and study selection

Relevant publications were identified by performing a comprehensive search across multiple databases, including PubMed, Web of Science, Cochrane Library, Proquest, Scopus, and Google Scholar. The search encompassed studies published over the past 10 years, up until January 31, 2024. The keywords used were “obstetric violence,” “obstetrical violence,” “disrespect,” “abuse during childbirth,” “respectful maternity care,” “childbirth,” “childbearing,” “maternity and care regulations,” “prevalence,” “rate,” “related factors,” “risk factors,” and “predisposing factors”. Specific search terms related to OBV and prevalence were used to ensure thoroughness; keywords were looked for in the titles and abstracts. To optimize the search results, we employed MeSH keywords and Boolean operators (such as AND, OR). The initial systematic search was conducted by two authors (SH and LA). The study selection process was facilitated by using the EndNote software (version X9, Thomson Reuters). The researchers MA and MO performed a comprehensive screening of the records retrieved from the literature search; they independently reviewed the titles and abstracts to identify potentially relevant publications. Subsequently, the full texts of the eligible publications were assessed.

Any disagreements in the screening process were resolved through group discussions involving a third senior researcher (SH). This collaborative approach ensured thorough resolution of any discrepancies in the interpretation or selection of publications. Finally, the full texts of the remaining articles were meticulously examined, and irrelevant studies were excluded based on predefined inclusion and exclusion criteria.

### 2.2 Eligibility criteria

The study was specifically focused on women who had given birth. The following criteria were established for study selection: research designs must employ an observational approach, and participants must be healthy women who had experienced childbirth, regardless of whether the delivery had been vaginal or via cesarean section. Research studies that did not include relevant data on OBV or disrespect experienced during childbirth, investigations that evaluated domestic violence, and those that solely relied on qualitative methods and did not provide quantitative data were excluded. Additionally, studies that had not undergone a peer‐review process, such as conference abstracts, editorials, or letters to the editor, were omitted. Studies classified as meta‐analyses or systematic reviews were also excluded.

### 2.3 Outcomes

The primary outcome was the rate of violence perpetrated against women during labor and childbirth by healthcare personnel.

The secondary outcome was a related factor or risk factors of OBV.

### 2.4 Data extraction

Data extraction was performed for each study using a predefined Excel form. The authors (LA, EC and NS) conducted the initial extraction process. To ensure precision and uniformity, two additional researchers (MO and MA) meticulously cross‐checked the extracted data. The data extraction form covered all key elements, including the paper's title, author, year of publication, country, study design, sampling strategy, study sample size, data collection questionnaires, the reported prevalence of OBV, its related subdomains (including sample and case numbers) and main results, as well as related factors of violence if any. Ultimately, 24 pertinent articles were included in the meta‐analysis phase.

### 2.5 Measurement

Violence during childbirth was assessed using a framework comprising seven dimensions of standards developed by the Maternal and Child Health Integrated Program (MCHIP) as well as set by Bowser and Hill.^22^ The forms of OBV based on Bowser and Hill considered in this study included physical abuse, non‐consented care, breaches of confidentiality, non‐dignified care, discriminatory care, abandoned or neglected care, and detention in health facilities.^23^ We also considered another classification of OBV divided into three dimensions: verbal, physical, and psycho‐affective^24^ (Table 1). After a careful assessment, the items categorized as verbal violence were found to be consistent with the dimension of non‐dignified care. However, a few studies measured OBV using variables that did not fit into the mentioned categories. In such cases, we determined them separately.

**TABLE 1:** Definition of obstetric violence.

Categories	Verification
Physical abuse	Pinching, slapping, pushing, beating, poking, rape, sexual harassment. Kicked, pinched, slapped, pushed, beaten, raped, tied to the delivery bed/delivery couch, or episiotomy without anesthesia.^39^, ^40^ Subjecting the woman to physical force (slapping, pushing, pinching, tying, or threats of beating), refusing pain control requests, forcing her to stay in an uncomfortable and painful position, cutting or suturing episiotomy incisions or perineal tears without anesthesia, applying fundal pressure (Kristeller maneuver), or restriction of oral fluid or food intake during labor without any explanation.^2^
Non‐consented care	No permission obtained before examination for medical procedures such as tubal ligation or hysterectomy.^37^ Abdominal palpation, vaginal examination, episiotomy, tubal ligation, or hysterectomy performed without the woman's consent. The woman's failure to obtain appropriate information about medical procedures from health professionals, her consent is not requested for medical procedures (cesarean section, episiotomy, augmentation of labor, vaginal examination, blood transfusion, enema, shaving, postpartum contraception or sterilization, or birth position), and her decision‐making power is limited.^37^
Non‐confidential care	HIV status shown to others; health information discussed with non‐health staff; physically exposed during delivery or examination; no screens blocking view during delivery or examination; discussed her issues when other clients were listening.^41^ HIV status was shown to others, other health information was shown to others, health information was discussed with non‐health staff, personal issues were discussed within earshot of others.^43^ Sharing personal details about the woman with persons who were not related to care or treatment, overcrowded examination/giving birth without privacy barriers such as curtains and drapes.^39^
Non‐dignified care	Use of non‐dignified language such as shouting and scolding; threats of withholding services/called insulting names, laughed at or scorned.^37^ Shouted at, scolded, threatened to withhold services, laughed at or scorned.^39^ Sarcastic attitude/rude treatment/imperative speech, shouting, scolding/insulting, offensive or derogatory remarks, being blamed for any reason, inhibiting emotional expression/not listening to what is said/ignoring questions about the birth process and the baby, and not permitting the birth companion to be present.^2^
Discriminatory care	Discrimination or ill‐treatment of a woman in the health facility where she is receiving childbirth care on any grounds, such as language, social status, economic status, appearance, belief, culture, or health status.^2^
Abandoned (neglected care)	Ignored when sought help for pain relief or left unattended by health workers when the woman needed help.^35^ Left unattended during labor, delivery, experiencing a complication, or after delivery when a service was needed.^39^ The woman not getting help for a long period of time, giving birth without a health professional, not getting the breastfeeding support she desires, or late intervention in any situation that might threaten her life.^2^
Detention at the healthcare facility	Detained when a woman is unable to pay for services.^2^ Kept in the health facility against her will.^39^ Detention of the woman for various reasons in the health facility providing childbirth care, demanding gifts or unofficial money from the woman/relatives.^2^
*Psycho‐affective violence*	
Corruption	Request for a bribe for services.^35^
Lack of privacy	Physically exposed during delivery or examination, or no screens blocking view during delivery or examination.^39^

### 2.6 Quality assessment

The quality of each study was assessed using a modified version of the Newcastle–Ottawa scale, as outlined by Stang.^25^ This modified version comprised five dimensions: sample representativeness, sample size, response rate, ascertainment of accuracy, and thoroughness of descriptive data reporting. Studies that were assigned a total Newcastle–Ottawa scale score of less than 3, indicating a high risk of quality, were excluded from the analysis.

### 2.7 Statistical analysis

The statistical software R (version 4.2.3) and the meta package were used for analysis. The metaprop command was used to calculate the pooled prevalence and its corresponding 95% confidence interval (CI). To minimize the effect of heterogenicity resulting from differences in the geographical location of studies, participants, and data collection questionnaires, we used the random effects model.

Heterogeneity across the studies was assessed using the *I* ^2^ statistic. Thresholds of ≥25%, ≥50%, and ≥75% were applied to low, moderate, and high heterogeneity, respectively.^26^ A forest plot was generated to provide a visual representation of the prevalence data from the studies. To determine factors related to OBV, risk factors were extracted from eligible studies. Meta‐regression analyses using the metafor command were performed to explore the potential association between OBV and study‐level variables.^27^ The random effects model was used to synthesize the effect size across studies. All the factors related to OBV were categorical or nominal; the odds ratio (OR) was used to assess the effect size. Publication bias was evaluated by visually inspecting the funnel plot. Additionally, Egger tests of publication bias were performed.^28^ The significance level was set to *P* < 0.05.

## 3 RESULTS

### 3.1 Characteristics of the analyzed studies

A total of 810 records were identified using our search strategy, with contributions from PubMed (689 records), Web of Science (53 records), Scopus (63 records), and Embase (5 records). Of these, 388 papers were duplicated, and 389 papers were irrelevant.

**FIGURE 1:**
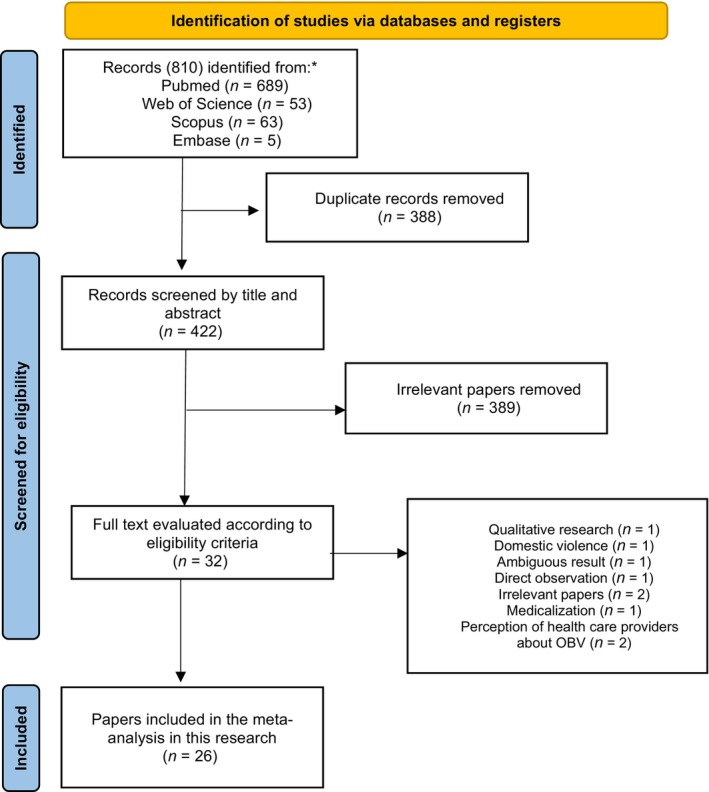
Flow chart of study.

The study selection process is shown in Figure 1. After a thorough review of the titles and abstracts, we identified 32 articles that were deemed relevant for full‐text analysis. However, upon further examination, seven studies were excluded. One was a qualitative study,^29^ and one was about domestic violence during pregnancy.^30^ One paper provided no clear definition of OBV, and one explored medicalization.^31^ Two studies examined the perception of medical students and healthcare providers about OBV.^31^, ^32^ Finally, one study reported the prevalence of OBV based on direct observation.^33^

The cumulative number of participants across these studies amounted to 57 119, and the studies were conducted in 15 nations. Of the 25 reports included in the analysis, four were derived from the American continent, four from Europe, eight from Africa, and seven from Asia. Notably, North and South America contributed the largest number, accounting for 47% of the total number of participants (28 894 individuals). With regard to research design, all but one of the included studies adopted a cross‐sectional and retrospective approach.^34^ Ten reports addressed OBV shortly after childbirth (up to 3 months),^35^, ^36^, ^37^, ^38^, ^39^, ^40^, ^41^, ^42^, ^43^, ^44^ and 16 papers addressed OBV in the long term (up to 27 years after childbirth).^3^, ^13^, ^17^, ^24^, ^31^, ^40^, ^45^, ^46^, ^47^, ^48^, ^49^, ^50^, ^51^, ^52^, ^53^

In 13 of 25 studies, the seven dimensions of neglected care, non‐confidential care, non‐consented care, non‐dignified care, discriminatory care, detention in health facilities, and physical abuse were examined.^3^, ^35^, ^36^, ^39^, ^41^, ^42^, ^44^, ^46^, ^47^, ^49^, ^50^, ^51^, ^54^ In three studies, the three dimensions of verbal violence, physical violence, and psycho‐affective violence were explored.^13^, ^24^, ^45^ Two studies reported violence based on two dimensions (violence and non‐consented care).^17^, ^48^ One study only reported factors related to OBV and did not address the magnitude of OBV among the participants.^34^

Table 2 provides a comprehensive summary of the characteristics of the 25 studies included in the meta‐analysis.

**TABLE 2:** Characteristics of the included studies.

Author year	Country	Sample size	Any form of violence	Neglected care	Non‐ consented care	Non‐ confidential care	Non‐dignified care	Detention in the health facility	Discrimination	Physical abuse	Sexual violence	Psycho‐affective	Repeated vaginal exam	Request for money	Episiotomy without consent	Kristeller maneuver	Forced to stay in a position	Lack of privacy
Yalley et al., 2023^50^	Ghana	1854	1210	619	139	663	528	142	204	508	—	—	—	—				
Ismail et al.^45^	Gaza	722	300							143	13	122						
Asci et al., 2023^3^	Turkey	513	392	228	132	17	129	0	2	228	—	—	—	—	—			
Azzam et al.^38^	Jordan	259							51	42					139			
Martinez‐Vázquez et al.^24^	Spain	583 (only women with an EPDS score <10)	357	—			106			287		160						
Molla et al.^47^	Ethiopia	661	527	53	436	152	268	0	20	145	17							
Scandurra et al.^48^	Italy	282	221		157		71									96	67	
Hajizadeh et al.^41^	Iran	334	253	64	126	222	275	3	304	212							148	112
Martínez‐Galiani et al., 2020^24^	Spain	899	606				226			490		330	200				123	
Mena‐Tudela et al., 2020^31^	Spain	16 383			2406		6045											
Castro et al.^17^	Mexico	24 064	8013		4115		2695										1708	
Mihret et al.^46^	Ethiopia	409	307	52	260	132	226	0	38	192								
Amole et al.^36^	Nigeria	332	171	142	75	114	87	17						12				
Tekele Bobo et al.^44^	Ethiopia	612	458	154	331	247	212	18	81	227								244
Brandão et al.^40^	Ecuador	252													30 (of 92)	49	62	
Wassihun et al.^49^	Ethiopia	410	275	29	236	45	35	5	9	236								34
Gebremichael et al.^52^	Ethiopia	1125	248	63		8	111			8							40	
Mesenburg et al.^43^	Brazil	4087	748	240			378			183								17
Ravaldi et al.^53^	Italy	424			34		48								52			
Bhattacharya et al.^51^	India	410	380	35		23	79	54		55				371				13
Hameed et al., 2018^54^	Pakistan	1334	1294	427	1080	920	420											
Lansky et al., 2017^42^	Brazil	555	70	11	38	3	34	0	3						36 (out of 285)	67 (out of 285)		
Banks et al., 2017^39^ (observed and reported cases were studied; we only considered reported cases)	Ethiopia	204	43	5	36	28	2	0		1						22		31
Abuya et al.^35^	Kenya	641		92	28	115	129	52		27				6				47
Asefa et al.^37^	Ethiopia	173	136	68	164	37	21		34	57							35	37

### 3.2 Prevalence of obstetric violence

**FIGURE 2:**
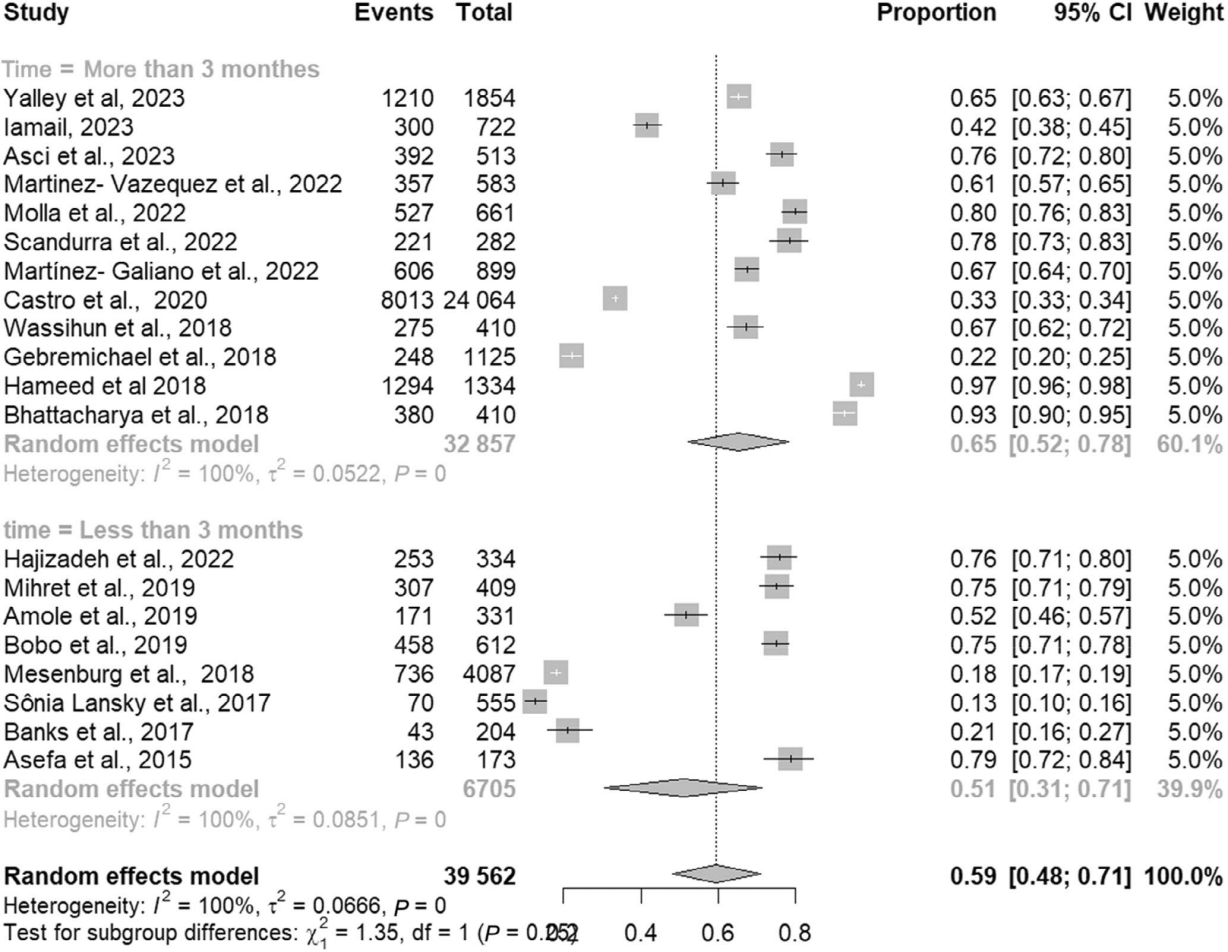
Forest plot of subgroup analysis.

The global prevalence of OBV estimated on the basis of 23 studies by the random effects model was 59% (95% CI 0.48–0.70; *I* ^2^ = 99.5%). The most prevalent subdomain of OBV was non‐consented care (37%; 95% CI 0.23–0.50; *I* ^2^ = 99.7%), while the least prevalent subdomain of OBV was detention in health facilities (0.02; 95% CI 0.00–0.03; *I* ^2^ *P* = 96.2%). The global pooled prevalence of OBV according to subdomains is summarized in Table 3, Figure 2.

**TABLE 3:** Worldwide pooled prevalence of OBV according to the subdomains of OBV.

OBV subdomain	Number of studies	OBV events	Sample size	Pooled prevalence (95%CI)	*I* ^2^ (%)	Tau^2^
Any type of OBV	23	15 997	39 565	0.59 (0.48–0.70)	99.5	2.48
Neglected care	16	2620	13 653	0.19 (0.12–0.26)	99.0	0.02
Non‐consented care	17	9793	49 085	0.37 (0.23–0.50)	99.7	0.07
Non‐confidential care	15	2726	9569	0.24 (0.14–0.35)	99.5	0.03
Non‐dignified care	22	12 172	56 291	0.24 (0.16–0.32)	99.7	0.03
Detention in the health facility	13	292	7107	0.02 (0.00–0.05)	96.2	0.00
Discriminatory care	11	917	6111	0.20 (0.02–0.38)	98.9	0.08
Physical abuse	18	3241	15 233	0.28 (0.18–0.37)	99.6	0.04
Sexual violence	2	30	1383	0.02 (0.013–0.028)	99.8	0.01
Psycho‐affective violence	3	612	2204	0.26 (0.15–0.38)	97.7	0.00
Request for money	3	389	1383	0.31 (0.10–0.86)	99.9	0.25
Episiotomy without consent	4	257	1060	0.27 (0.07–0.47)	96.9	0.03
Kristeller maneuver	4	234	1027	0.21 (0.12–0.30)	93.1	0.003
Forced to stay in a position	7	2183	27 129	0.19 (0.09–0.29)	98.4	0.01
Lack of privacy	9	1070	13 682	0.15 (0.06–0.24)	98.7	0.01

Abbreviations: CI, confidence interval; OBV, obstetric violence.

### 3.3 Subgroup analyses

As the time periods after childbirth investigated in the various studies were diverse (1 h to 27 years), we performed a subgroup analysis and calculated the pooled prevalence of OBV among studies that had measured violence up to 12 weeks after childbirth; the results are shown in Table 3. The prevalence of any type of OBV was 55% (95% CI 0.43–0.70). The most prevalent type of OBV was discriminatory care 36% (95% CI 0.25–0.67); the details are shown in Table 4.

**TABLE 4:** Subgroup analysis of OBV by time of questionnaire completion (up to 12 weeks post‐delivery).

OBV subdomain	Number of studies	OBV events	Sample size	Pooled prevalence (95% CI)	*I* ^2^ (%)	Tau^2^
Any type of OBV	8	2174	6705	0.55 (0.43–0.70)	99.5	2.48
Neglected care	8	814	6937	0.21 (0.10–0.31)	87.4	0.02
Non‐consented care	7	798	2850	0.34 (0.10–0.57)	99.8	0.07
Non‐confidential care	8	774	3975	0.24 (0.09–0.39)	99.4	0.04
Non‐dignified care	8	1138	6937	0.23 (0.05–0.41)	99.6	0.03
Detention in the health facility	7	91	2850	0.023 (0.00–0.04)	93.4	0.001
Discriminatory care	6	644	2264	0.36 (0.25–0.67)	99.8	0.12
Physical abuse	7	749	6310	0.20 (0.01–0.39)	99.2	0.05
Request for money	4	389	1995	0.31 (0.10–0.86)	99.9	0.25
Episiotomy without consent	3	205	636	0.32 (0.09–0.56)	97.2	0.04
Kristeller maneuver	3	138	741	0.17 (0.10–0.25)	87.1	0.003
Forced to stay in a position	7	245	759	0.29 (0.15–0.44)	95.2	0.01
Lack of privacy	6	488	6051	0.19 (0.07–0.31)	88.4	0.01

*Note*: Studies included in the analysis: Abuya et al.^35^; Amole et al.^36^; Asefa et al.^37^; Azzam et al.^38^; Banks et al.^39^; Brandão et al.^40^; Hajizadeh et al.^41^; Lansky et al.^42^; Mesenburg et al.^43^; Tekele Bobo et la.^44^

Abbreviations: CI, confidence interval; OBV, obstetric violence.

### 3.4 Risk factors for any type of obstetric violence

Briefly, 26 studies were evaluated to determine factors related to OBV. The elicited factors were entered into a meta‐regression model. Table 4 shows the results of the meta‐regression. Among the related factors, the following were not significantly associated with OBV: place of delivery (public hospital vs. private hospital, *P* = 0.394), parity (primiparous vs. multiparous, *P* = 0.421), educational status of women (uneducated vs. educated mother with maximum 9 years of education, *P* = 0.191), complicated childbirth (complicated vs. non‐complicated, *P* = 0.101), and age of women (younger than 19 years vs. older than 19 years, *P* = 0.211).

However, the following factors were found to be significantly associated with OBV: the presence of a midwife as skilled personnel beside the woman during childbirth [OR (95% CI) = 0.4 (0.2–0.9)] is liable to reduce the likelihood of OBV; middle and high levels of income [OR (95% CI) = 0.7 (0.7–0.9)] might also reduce the likelihood of OBV; and vaginal delivery [OR (95% CI) = 2.08 (1.1–3.08)] is liable to increase the likelihood of OBV (Table 5).

**TABLE 5:** Meta‐regression of factors related to OBV based on the random model.

Risk factor	Number of included studies	OR	95% CI	*I* ^2^	*P*‐value
Public hospital versus government hospital	5	1.27	0.7–2.2	0.78	0.394
Midwife as attendant during labor and delivery versus doctor/obstetric resident	4	0.4	0.2–0.9	0.23	0.021
Primiparous versus multiparous	5	1.01	0.7–1.6	0.97	0.421
Vaginal delivery versus Cs	4	2.08	1.1–3.8	0.87	0.012
Educated (maximum 9 years) versus uneducated	11	0.8	0.5–1.9	0.89	0.191
High and mid‐level income versus low level	7	0.5	0.2–0.7	0.23	0.006
Having complications during delivery versus uncomplicated delivery	3	1.1	0.2–5.5	0.99	0.101
Age ≥19 versus age <19	12	1.3	0.7–6.9	0.98	0.211

Abbreviations: CI, confidence interval; OBV, obstetric violence; OR, odds ratio.

### 3.5 Risk of bias assessment

Eight studies were of good quality, 16 of fair quality, and one of poor quality; all of these were included in the analysis (Table 6).

**TABLE 6:** Critical appraisal of the studies.

	Study	Selection				Comparability	Outcome		Score	Quality
1	Yalley et al.^50^	1‐a*	2‐a*	3‐a*	4‐c	1‐a**	1‐d	2‐a*	6	Fair
2	Ismail et al.^45^	1‐a*	2‐a*	3‐a*	4‐c	1‐a**	1‐d	2‐a*	6	Fair
3	Aşci and Bal. 2023^2^	1‐a*	2‐a*	3‐a*	4‐c	1‐a**	1‐d	2‐a*	6	Fair
4	Azzam et al.^38^	1‐c	2‐c	3‐c	4‐c	1‐a**	1‐d	2‐a*	3	Fair
5	Martinez‐Vázquez et al.^24^	1‐a*	2‐a*	3‐b	4‐c	1‐a**	1‐d	2‐a*	5	Fair
6	Molla et al.^47^	1‐b*	2‐a*	3‐a*	4‐c	1‐a**	1‐c*	2‐a*	7	Good
7	Scandurra et al., 2021^48^	1‐d	2‐b	3‐c	4‐c	1‐a**	1‐c*	2‐a*	4	Fair
8	Hajizadeh et al.^41^	1‐a*	2‐a*	3‐a*	4‐c	1‐a**	1‐c*	2‐a*	7	Good
9	Martínez‐Galiano et al., 2020^13^	1‐a*	2‐a*	3‐a*	4‐c	1‐a**	1‐d	2‐a*	6	Fair
10	Mena‐Tudela et al., 2020^31^	1‐d	2‐b	3‐c	4‐c	1‐b	1‐d	2‐a*	1	Poor
11	Castro et al.^17^	1‐a*	2‐a*	3‐b	4‐c	1‐a**	1‐c*	2‐b	5	Fair
12	Mihret et al.^46^	1‐a*	2‐a*	3‐a*	4‐c	1‐a**	1‐c*	2‐a*	7	Good
13	Amole et al.^36^	1‐a*	2‐a*	3‐a*	4‐c	1‐a**	1‐c*	2‐a*	7	Good
14	Brandão et al.^40^	1‐a*	2‐a*	3‐a*	4‐c	1‐b	1‐d	2‐a*	4	Fair
15	Tekele Bobo et al.^44^	1‐a*	2‐a*	3‐b	4‐c	1‐a**	1‐c*	2‐a*	6	Fair
16	Wassihun et al.^49^	1‐a*	2‐a*	3‐a*	4‐c	1‐a**	1‐c*	2‐a*	7	Good
17	Goli et al., 2018^34^	1‐a*	2‐a*	3‐a*	4‐c	1‐a**	1‐d	2‐b	5	Fair
18	Gebremichael et al.^52^	1‐a*	2‐b	3‐a*	4‐c	1‐a**	1‐c*	2‐a*	6	Fair
19	Hameed et al.^54^	1‐a*	2‐a*	3‐a*	4‐c	1‐a**	1‐c*	2‐a*	7	Good
20	Mesenburg et al.^43^	1‐c	2‐c	3‐c	4‐c	1‐a**	1‐d	2‐a*	3	Fair
21	Bhattacharya et al.^51^	1‐a*	2‐b	3‐a*	4‐c	1‐a**	1‐c*	2‐a*	6	Fair
22	Lansky et al., 2017^42^	1‐b*	2‐b	3‐b	4‐c	1‐a**	1‐d	2‐a*	4	Fair
23	Banks et al., 2017^39^	1‐a*	2‐a*	3‐a*	4‐c	1‐a**	1‐c*	2‐a*	7	Good
24	Abuya et al.^35^	1‐a*	2‐a*	3‐a*	4‐c	1‐a**	1‐c*	2‐a*	7	Good
25	Asefa et al.^37^	1‐b*	2‐a*	3‐b	4‐c	1‐b	1‐c*	2‐a*	4	Fair

**FIGURE 3:**
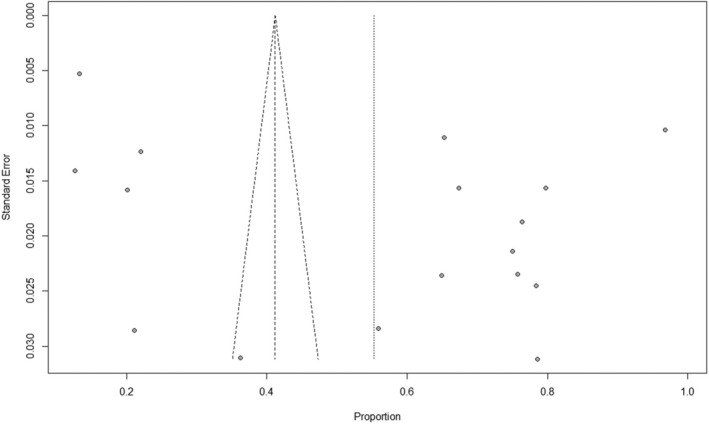
Funnel plot of the included studies.

Potential publication bias was checked via examination of funnel plots (Figure 3). The plot appears to be asymmetrical, with a gap or lack of studies on the left side of the funnel, particularly in the lower portion. This asymmetry suggests the possibility of publication bias: small studies with low prevalence estimates might have been less likely to be published or included in the meta‐analysis.

## 4 DISCUSSION

The meta‐analysis revealed a global prevalence of 60% for OBV, with substantial variations across the studies. This high prevalence rate indicates that OBV is a pervasive issue, affecting a significant proportion of women during childbirth. The most common identified form of OBV was non‐consented care (37%), reflecting issues related to autonomy and informed consent in obstetric care. Conversely, detention in health facilities was the least prevalent form (2%), suggesting that while this practice is relatively rare, it still constitutes a significant violation of human rights where it occurs.

The high rate of non‐consented care might be due to several factors. Healthcare providers might not be fully aware of the importance of obtaining informed consent from patients. They might not regard the non‐consented practices as significant contributors to mistreatment.^55^ They might view these behaviors as routine or necessary medical interventions, without recognizing the patient's perspective. Non‐consented behaviors might have become normalized within the healthcare system.^56^

The meta‐regression analysis identified several risk factors associated with a high rate of OBV. Notably, vaginal delivery was associated with a greater likelihood of experiencing OBV compared to cesarean section. This could be due to the longer time period and interaction required during vaginal delivery.^57^, ^58^ The latter is a more prolonged and hands‐on process than cesarean section. The longer time period and close contact might create more opportunities for inappropriate, disrespectful, or abusive behaviors referred to as OBV.

The presence of a midwife during childbirth was associated with a lower likelihood of OBV, suggesting that midwife‐led care models might offer a more respectful and supportive birthing experience. Midwives typically spend more time with the laboring woman, fostering a collaborative relationship and ensuring informed consent. Midwives are less likely to resort to unnecessary interventions, which might then become a source of OBV. Moreover, midwives usually provide care throughout pregnancy, labor, and in the postpartum period, creating a trusting relationship.^59^, ^60^

A higher economic status was also correlated with a lower risk of OBV, highlighting socioeconomic disparities in maternal care. Evidence has shown that individuals with a higher income might have better access to healthcare providers and facilities that prioritize patient‐centered, high‐quality care.^61^ They have greater health literacy and awareness of their rights as patients.^62^ Quite possibly, affluent persons have more leverage in demanding respectful treatment and resisting abusive or coercive practices.

The studies included in the meta‐analysis spanned 14 countries, illustrating the global nature of OBV. The large number of regions underlines the fact that OBV is not confined to a single geographical area requiring targeted attention. However, the variations in the prevalence of OBV across regions also suggest that local cultural, systemic, and healthcare factors play a significant role in shaping these experiences. In the EU countries, non‐consented care is more prevalent than non‐dignified care. One of the likely reasons is that OBV is recognized as a violation of human rights in EU countries and is a widely debated subject among healthcare policymakers.^55^, ^56^

Pregnancy and childbirth place a woman in a vulnerable state both mentally and physically.^63^ OBV might have serious harmful effects on a mother's mental and even physical health.^12^ Effective pre‐service and in‐service educational programs must be incorporated into the curriculum of healthcare providers, especially midwives, obstetricians, and obstetric residents. Healthcare providers working in the obstetric ward, in direct contact with mothers, should be held accountable in every case of OBV.

The limitations of the present study are worthy of mention. The first limitation was the absence of a standardized tool and universally accepted definition of OBV. The next limitation was the existence of overlapping sub‐categories of OBV. Additionally, the data of the present study were primarily derived from women's experiences, which might lead to an overestimation or underestimation of the prevalence of OBV. As most studies included in the meta‐analysis were retrospective in design, the potential for recall bias must be taken into account.

Furthermore, the significant heterogeneity observed in our meta‐analysis, with an *I* ^2^ of 99.5%, reflects variability in population characteristics, study design, and duration of follow‐up. While we conducted subgroup analyses based on the timing of questionnaire completion, we faced limitations due to the scarcity of studies from high‐income countries, with only five papers from Italy and Spain. This lack of data restricted our ability to comprehensively explore differences based on healthcare systems and cultural contexts. Additionally, factors such as healthcare provider training, institutional policies, and healthcare inequalities were not fully addressed in the included studies, potentially contributing to the observed heterogeneity. These limitations highlight the need for more standardized research methodologies in future studies on OBV.

## 5 RESEARCH RECOMMENDATIONS

Future research should include longitudinal investigations to comprehend the evolution and long‐term effects of OBV on women's health and well‐being. The lack of standardized definitions and measurements of OBV in the analyzed studies highlights the need for a unified framework. Developing and adopting standardized tools for assessing OBV will facilitate more accurate and comparable research. Research should focus on evaluating the effectiveness of various interventions aimed at reducing OBV, which would encompass the effect of midwife‐led care models, informed consent protocols, and cultural competence training for reducing OBV.

## 6 CONCLUSION

This systematic review and meta‐analysis underscored the prevalence and multifaceted nature of OBV, which concerns women across diverse global settings. The findings highlight the urgent need for interventions at multiple levels (i.e., healthcare practice, policy, and research) to address this pervasive issue. Prioritizing respectful maternity care, enhancing informed consent, supporting midwife‐led care, and addressing socioeconomic disparities will help in evolving a maternal healthcare system that ensures safety, dignity, and respect for all women.

## AUTHOR CONTRIBUTIONS

Conception: Sevil Hakimi and Ibrahim Alkatout. Design: Sevil Hakimi, Ibrahim Alkatout and Esin Ceber Turfan. Systematic search: Sevil Hakimi, Leila Allahqoli, Maryam Alizadeh andMeryem Ozdemir. Data Extraction: Leila Allahqoli, Esin Ceber Turfan, Neriman Sogukpinar. Quality assessment: Meryem Ozdemir, Hamid Soori, Neriman Sogukpinar. Statistical Analysis: Sevil Hakimi and Leila Allahqoli. Manuscript draft: Sevil Hakimi. Ibrahim Alkatout. Hamid Soori. Manuscript editing: all authors. Manuscript review: all authors. Final approval of manuscript: all authors.

## FUNDING INFORMATION

The authors did not receive any financial support for this work, and no funding was obtained for conducting this study.

## CONFLICT OF INTEREST STATEMENT

The authors declare that, there is no conflict of interest.

## Supporting information


**Data S1:** Supporting Information.

## Data Availability

Research data are not shared.
